# The Identification of the Biomarkers of Sheng-Ji Hua-Yu Formula Treated Diabetic Wound Healing Using Modular Pharmacology

**DOI:** 10.3389/fphar.2021.726158

**Published:** 2021-11-16

**Authors:** Jing-Si Jiang, Ying Zhang, Ying Luo, Yi Ru, Yue Luo, Xiao-Ya Fei, Jian-Kun Song, Xiao-Jie Ding, Zhan Zhang, Dan Yang, Shuang-Yi Yin, Hui-Ping Zhang, Tai-Yi Liu, Bin Li, Le Kuai

**Affiliations:** ^1^ Department of Dermatology, Yueyang Hospital of Integrated Traditional Chinese and Western Medicine, Shanghai University of Traditional Chinese Medicine, Shanghai, China; ^2^ Institute of Dermatology, Shanghai Academy of Traditional Chinese Medicine, Shanghai, China; ^3^ Shanghai Skin Disease Hospital of Tongji University, Shanghai, China; ^4^ Center for Translational Medicine, Huaihe Hospital of Henan University, Kaifeng, China; ^5^ Shanghai Applied Protein Technology Co., Ltd., Shanghai, China

**Keywords:** diabetic wound healing, SJHY formula, modular pharmacology, molecular docking, experimental verification

## Abstract

Sheng-Ji Hua-Yu (SJHY) formula has been proved to reduce the severity of diabetic wound healing without significant adverse events in our previous clinical trials. However, based on multi-target characteristics, the regulatory network among herbs, ingredients, and hub genes remains to be elucidated. The current study aims to identify the biomarkers of the SJHY formula for the treatment of diabetic wound healing. First, a network of components and targets for the SJHY formula was constructed using network pharmacology. Second, the ClusterONE algorithm was used to build a modular network and identify hub genes along with kernel pathways. Third, we verified the kernel targets by molecular docking to select hub genes. In addition, the biomarkers of the SJHY formula were validated by animal experiments in a diabetic wound healing mice model. The results revealed that the SJHY formula downregulated the mRNA expression of *Cxcr4*, *Oprd1*, and *Htr2a*, while upregulated *Adrb2*, *Drd*, *Drd4*, and *Hrh1*. Besides, the SJHY formula upregulated the kernel pathways, neuroactive ligand–receptor interaction, and cAMP signaling pathway in the skin tissue homogenate of the diabetic wound healing mice model. In summary, this study identified the potential targets and kernel pathways, providing additional evidence for the clinical application of the SJHY formula for the treatment of diabetic wound healing.

## 1 Introduction

Diabetic wounds are a severe health problem, while the prevalence of diabetes worldwide and foot ulceration is approximately 3% in a community-based cohort clinical study (Zhang et al., 2017; Narres et al., 2017). It is estimated that up to 50% of patients with diabetic foot ulceration have peripheral arterial disease in high- or middle-income countries (Prompers et al., 2007; Morbach et al., 2012), whereas a higher incidence of neuropathic ulcers was observed in low-income countries (Rigato et al., 2018; Younis et al., 2018). Chronic ulcers, resulted in amputation, are among the most frequent complications corresponding to diabetes, which account for over 200 billion USD in annual in healthcare costs (den Dekker et al., 2019).

In the field of clinical treatment, platelet-derived growth factor was the only drug approved by U.S. Food and Drug Administration for the treatment of diabetic wound to date. However, availability of it was limited due to heavy cost (Papanas and Maltezos, 2007; Barrientos et al., 2008). As recommended in guidelines (Guo et al., 2020), arterial reconstruction of the lower limbs is the moose vital measure, while patients undergoing surgical revascularization are at increased risk of surgical mortality (Hinchliffe et al., 2016). Consequently, appropriate interventions targeted as diabetic wound should be taken to improve long-term clinical efficacy.

The clinical application and research of traditional Chinese medicine (TCM)–treated diabetic wound healing have been extensively investigated and broadly applied. The SJHY formula is an effective compound preparation of Chinese herbal medicine for treating diabetic wounds, which was formulated on the basis of TCM theories of “qu-fu-sheng-xin (removing necrotic tissues to stimulate the growth of new skin)” (Li et al., 2012) and “sheng-ji-hua-yu (promoting the growth of new tissues and simultaneously melting the stasis)” (Li et al., 2011). SJHY formula is summed up according to clinical practice, while consisting of Sheng-ji recipe and Hua-yu recipe (Li, 2000). Herbal formula treatment is efficient in the management of diabetic skin ulcers, and healing time is 2–3 days shorten than western medicine treatment (mupirocin ointment, growth factor (bFGF), and Vaseline gauze for external use and with basic therapies), which are consistment with our previous animal experiment reported (Li et al., 2011; Kuai et al., 2018; Xiang et al., 2021). Additionally, the SJHY formula has been validated to be efficacious to treat diabetic wound healing *via* higher order Markov chain set pair analysis (Kuai et al., 2020) and cloud model set pair analysis (Kuai et al., 2019). However, further investigations are required to elucidate the regulation mechanisms of SJHY formula–treated diabetic wound healing.

The effectiveness of single-target therapy is inadequate for complex diseases recently (Lord and Ashworth, 2010). Due to the characteristics of complexity of diseases with multiple targets, modular pharmacology, which employed multiple modules according to combination therapy, was developed, which has been considered to be the next paradigm in drug discovery (Wang et al., 2012). Taking into consideration topological overlap, ClusterONE can improve the performance of module detection, together with an effect in predicting functional modules (Cheng et al., 2019; Amjad et al., 2020). Hence, the approach of modular pharmacology using the ClusterONE tool has been used for research on TCM and natural medicine (Chen et al., 2016).

Molecular docking, a computational technique extensively adopted in drug discovery, which established *in silico* structure-based docking enables predicting bond conformations and free binding energies, and places small molecule ligands to targets predicting ligand–target interactions at a molecular level (Pinzi and Rastelli, 2019).

**FIGURE 1:**
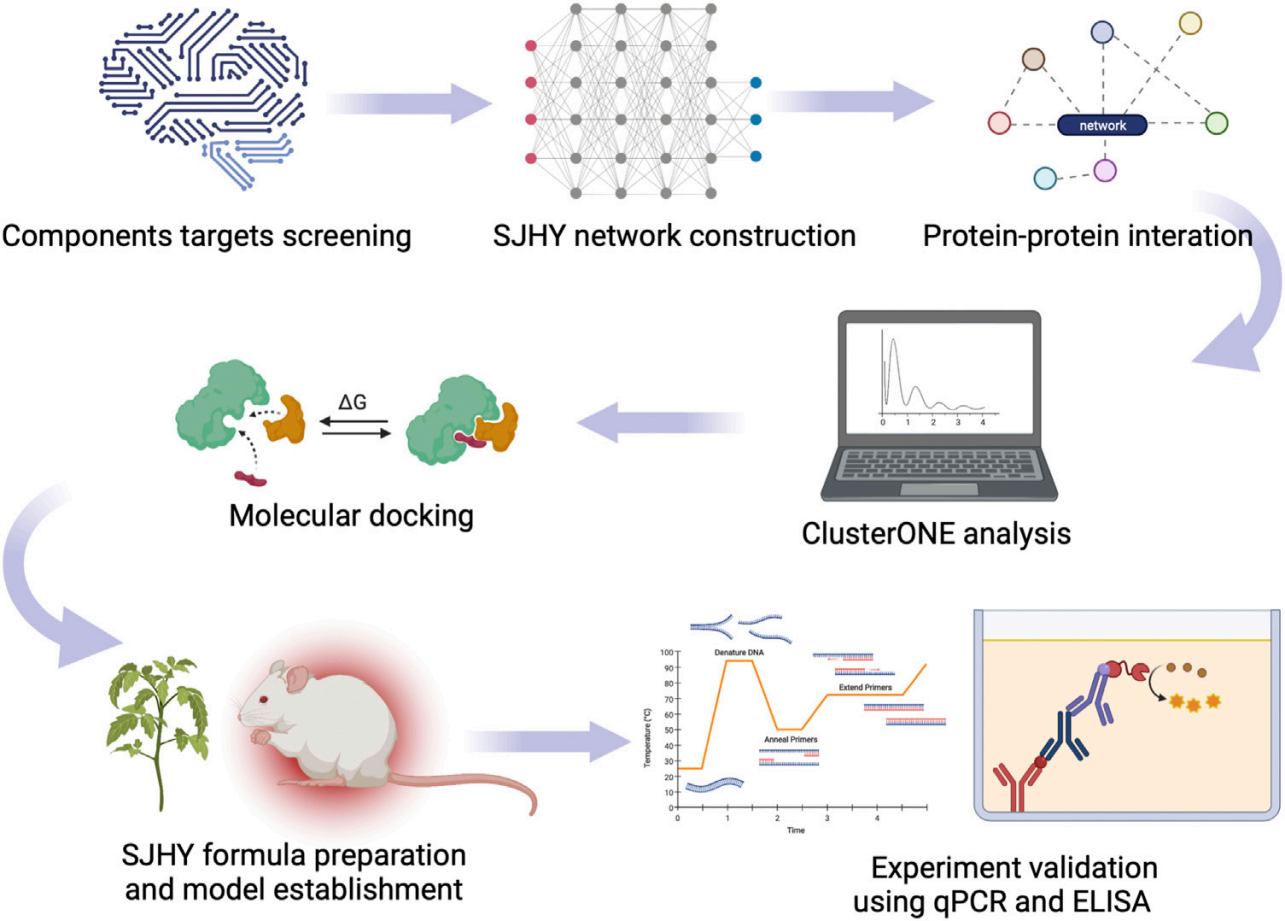
Study design.

Based on the evidences, the current studies applied the ClusterONE algorithm for modular pharmacology to identify biomarkers of SJHY formula on the treatment of diabetic wound healing. Furthermore, we verified the targets by molecular docking and experiments *in vivo* (Figure 1).

## 2 Methods

### 2.1 Components and Corresponding Targets of SJHY Formula

The search was performed on TCMSP (https://www.tcmspw.com, Ru et al., 2014), the comprehensive database of TCMID (http://www.megabionet.org/tcmid/, Huang et al., 2018), and BATMAN-TCM (http://bionet.ncpsb.org/batman-tcm, Liu et al., 2016), using “Huang Qi,” “Dan Shen,” “Bai Zhi,” “Da Huang,” “Zi Cao,” “Xue Jie,” “Lu Gan Shi,” and “Zhen Zhu Fen” as keywords. Table 1 shows *Latin* name or English name of each herb. Information of molecular molecule IDs, molecule names, and target names for each herb has been downloaded. The gene names of targets were largely annotated on the UniProt database (https://www.uniprot.org), while subsequently normalized to gene symbol.

**TABLE 1:** Number of components and potential targets of SJHY formula.

Herbal name	*Latin* name or English name	Number of components
Huang Qi	*Astragalus mongholicus* Bunge, root	65
Dan Shen	*Salvia miltiorrhiza* Bunge., root and rhizome	164
Bai Zhi	*Angelica dahurica* (Fisch. ex Hoffm.) Benth. et Hook. f. ex Franch. et Sav	193
Da Huang	*Rheum palmatum* L	60
Zi Cao	*Lithospermum erythrorhizon* Sieb. et Zucc	37
Xue Jie	*Daemonorops draco* Bl	5
Lu Gan Shi	(Zn2+.2 [Fe3+].4 [O2-])	2
Zhen Zhu Fen	Pearl powder	4

### 2.2 Acquisition Targets of Diabetic Wound Healing

Corresponding targets associated with the disease of diabetic wound healing were collected from the GeneCards database (https://www.genecards.org, Lin and Hu, 2021), Online Mendelian Inheritance in Man database (OMIM, https://www.omim.org, Amberger and Hamosh, 2017), PharmGKB database (https://www.pharmgkb.org, Barbarino et al., 2018), Therapeutic Targets Database (TTD, db.idrblab.net, Wang Y. et al., 2020), and DrugBank database (https://www.drugbank.com, Wishart et al., 2018). An intersection of targets associated with diabetic wound healing diseases and the targets of SJHY formula was constructed, as potential targets for subsequent gene annotation and an enrichment analysis.

### 2.3 Component–Target Network Construction

According to the pairs of herbs components and component–target, the SJHY formula network image was produced using Cytoscape software (version 3.6.0). Nodes of different colors represented core ingredients or targets, and the edges were used to describe the interaction relationship between each Chinese medicine and the ingredients along with the targets.

### 2.4 Gene Annotation and Function Enrichment

In the current study, the gene annotation and the function enrichment analysis were performed using Metascape (http://metascape.org) (Zhou et al., 2019), which consist of the Gene Ontology (GO) function enrichment analysis, including the biological process, cellular component, molecular function, reactome gene sets, canonical pathways, CORUM, and Kyoto Encyclopedia of Genes and Genomes (KEGG). A *p*-value < 0.01 and an enrichment score of >1.5 were considered to be statistically significant.

### 2.5 Protein–Protein Interaction Network

Protein–protein interaction (PPI) networks are used as viable tools for understanding disease mechanisms, drug discovery, or repositioning drugs (Vella et al., 2018). The STRING database (https://string-db.org/) was particularly adept at collection, scoring, and integrating all publicly available sources. The present study conducted a PPI network study based on potential targets of SJHY formula and diabetic wound healing to investigate the interactions with the highest confidence (0.900). Furthermore, the network analyzer of network topology parameters was used as a tool to calculate the average degree of freedom, and the PPI data was obtained for further modular analysis.

### 2.6. Modular Analysis Using ClusterONE Algorithm

The ClusterONE algorithm aims to calculate the topology graph of the PPI network (Chen et al., 2016) and identifies modules in protein interaction networks (Cheng et al., 2019). The determined clusters were considered as kernel targets, which were defined as the node size >5, *p*-value < 0.0001, quality >0.60, and density >0.05.

Next, genes were sorted according to the degree of freedom in each inner cluster, and we identified three markers per cluster in the modular-based approach as hub genes of SJHY formula for the treatment of diabetic wound healing. Furthermore, we constructed a target pathway network map based on kernel clusters, and selected kernel pathways according to the size of nodes. Furthermore, the kernel targets of each cluster were used for the GO network and the KEGG target network analysis.

### 2.7 Molecular Docking Verification

We downloaded the compound SDF structure from the PubChem database (https://pubchem.ncbi.nlm.nih.gov/, Kim et al., 2021), used Chem3D energy minimization to save it in mol2 format, and AutodockTools 1.5.6 to open the small ligand molecule and saved it as a pdbqt file. Furthermore, we selected human-derived protein with a co-crystal structure and high resolution as the protein used for docking in this subject from Uniprot (https://www.uniprot.org/, The UniProt Consortium. 2017). The three-dimensional structures of the protein were downloaded from the RCSB database (www.rcsb.org/, Burley et al., 2018). AutodockTools 1.5.6 was adopted to add all hydrogen atoms, calculated Gasteiger charge, defined as an acceptor, and saved as a pdbqt file. Autodock Vina was used for semi-flexible docking. The lowest docking energy was considered as a condition to select for the docking binding mode analysis, the force was analyzed using Discovery Studio, and Pymol was used for mapping.

### 2.8 Experimental Verification

#### 2.8.1 SJHY Formula Preparation

SJHY formula consists of eight Chinese herbs (Supplementary Table S1). The herbs of SJHY formula were approved and obtained from the pharmacy department. The preparation process of SJHY formula was in accordance with our previous study (Kuai et al., 2018). The effective components of SJHY formula were extracted with 95% ethanol and mixed with carbomer. The mixture was stored at 4°C. For the LC-MS/MS analysis, the SJHY formula (50 μl) was first diluted with 40% methanol solution (950 μl, pre-cooled) and labeled as di20. Then we adulterated the di20 (50 μl) with 40% methanol solution (450 μl, pre-cooled) and stamped it as di200. All the diluted samples were centrifuged at 4°C at 16,000 rpm for 15 min, and the supernatant was transferred to the liquid vial and stored in the refrigerator at 4°C.

#### 2.8.2 Liquid Chromatography Tandem–Mass Spectrometer (LC-MS/MS)

LC-MS/MS analysis was performed for quality control of SJHY formula utilizing Waters ACQUITY UPLC I-Class coupled with a 5500 QTRAP mass spectrometer (SCIEX) at the column temperature of 35°C. The mobile phase (A: aqueous solution containing 0.1% formic acid; B: acetonitrile solution containing 0.1% formic acid) was delivered at a speed of 0.3 ml/min: 0–3 mins (5%–25%B); 3–8.5 mins (25%–45%B); 8.5–12 mins (45%–95%B); 12–15 mins (95%–98%B); 15–15.2 mins (98%–5%B); and 15.2–18.2 mins (5%B).

#### 2.8.3 Animals

The current study used C57BL/6 mice with the specific pathogen-free (SPF), placed at a temperature of 21–25°C for 16 h and dark for 8 h, provided by the Shanghai Medical Experimental Animal Center. The experiment was approved by the Ethics Committee of Yueyang Hospital (No.19964, Supplementary Figure S1). The mice were distributed into three groups (*n* = 4 for each group) randomly, as follows: control group, disease group, and SJHY group.

#### 2.8.4 Model Establishment

A diabetic skin wound mice model was established in the current study. Animals in the disease group and SJHY group were intraperitoneally injected with 2% streptozotocin (dissolved in a citrate buffer solution). The condition of the diabetic model was blood glucose (BG) over 16.7 mmol/L. After shaving, a sterile punch was adopted to make full-thickness excisional skin wounds (Kuai et al., 2018; Xiang et al., 2021). The intervention measures of the disease group and the control group were vehicle ointment, while SJHY ointment was applied to the SJHY group. The mice were executed and skin tissues were taken on day 9 after punching.

#### 2.8.5 qPCR

Total RNA was isolated from mouse skin tissues with a Trizol reagent (Invitrogen, USA). Real-time PCR was performed by the FastStart Universal SYBR Green Master (Rox) (Roche). Non-specific amplifications have been monitored using melting curves. The relative expression level was calculated according to the 2-ΔΔCt method. PCR primer sequences are displayed in Supplementary Table S2.

#### 2.8.6 ELISA

ELISA was performed using mice skin tissue. The conditions include incubation at 4°C and centrifugation for 5 min. cAMP mouse ELISA kit (CSB-E15061m, CUSABIO) and AGTR1 mouse ELISA kit (LS-F18244, LSBio) were used to monitor cAMP and AGTR1 protein content.

#### 2.8.7 Statistical Methods

Data involved in experimental validation was analyzed with GraphPad Prism 8. The T test was produced to compare among the groups, and a *p*-value of less than 0.05 was used as the condition for statistically significant difference.

## 3 Results

### 3.1 Identification of SJHY Formula and Disease Targets

**FIGURE 2:**
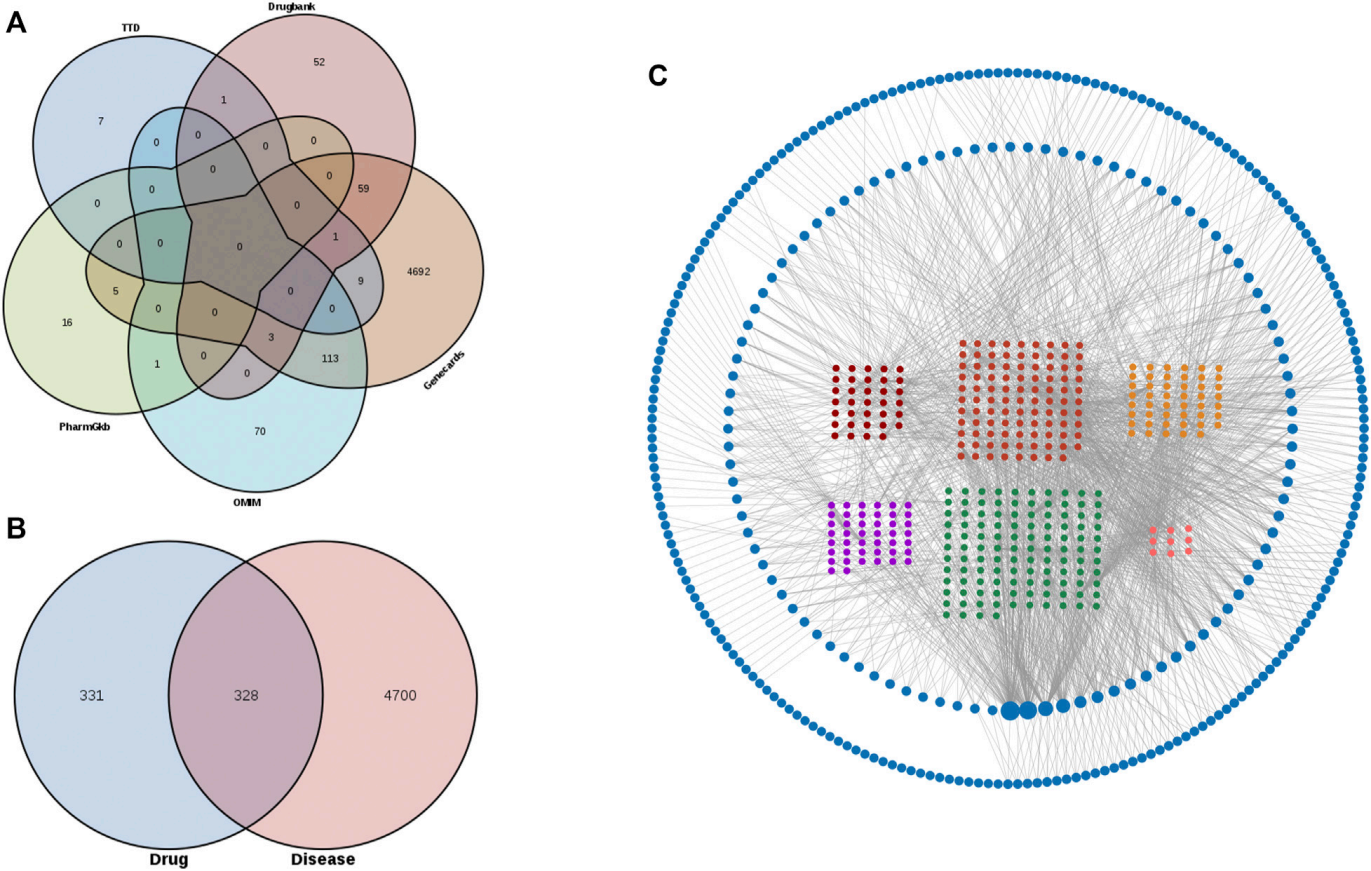
Potential targets and the enrichment analysis of diabetic wound healing and SJHY formula identification. **(A)** Venn graph of potential targets of diabetic wound healing using five databases. **(B)** Venn graph of the intersection of diabetic wound healing and SJHY formula potential targets. **(C)** A network of components and targets construction.

To obtain compound disease common targets, we retrieved on the TCMSP, TCMID, BATMAN-TCM, GeneCards, OMIM, PharmGKB, TTD, and DrugBank databases. 529 components were obtained, and 659 SJHY formula corresponding targets of these components were retrieved after screening on the TCMSP database (Supplementary Table S3). 5,028 genes associated with diabetic wound healing were collected from GeneCards, OMIM, PharmGKB, TTD, and DrugBank databases. After the restriction of conditions (by intersecting the targets associated with diabetic wound healing diseases and the targets of SJHY formula), 328 targets were identified (Figures 2A,B).

### 3.2 Component–Target Network Construction

To clarify how the SJHY formula may act against diabetic wound healing, we used Cytoscape software to build an ingredient–target network (Figure 2C; Supplementary Table S4). A node represented each protein, and the node’s size was proportional to the node’s degree. The edges denoted that nodes could interact with each other. Here, 328 targets after intersection were included in the compound–target interaction network.

### 3.3 Gene Annotation and Enrichment Analysis

**FIGURE 3:**
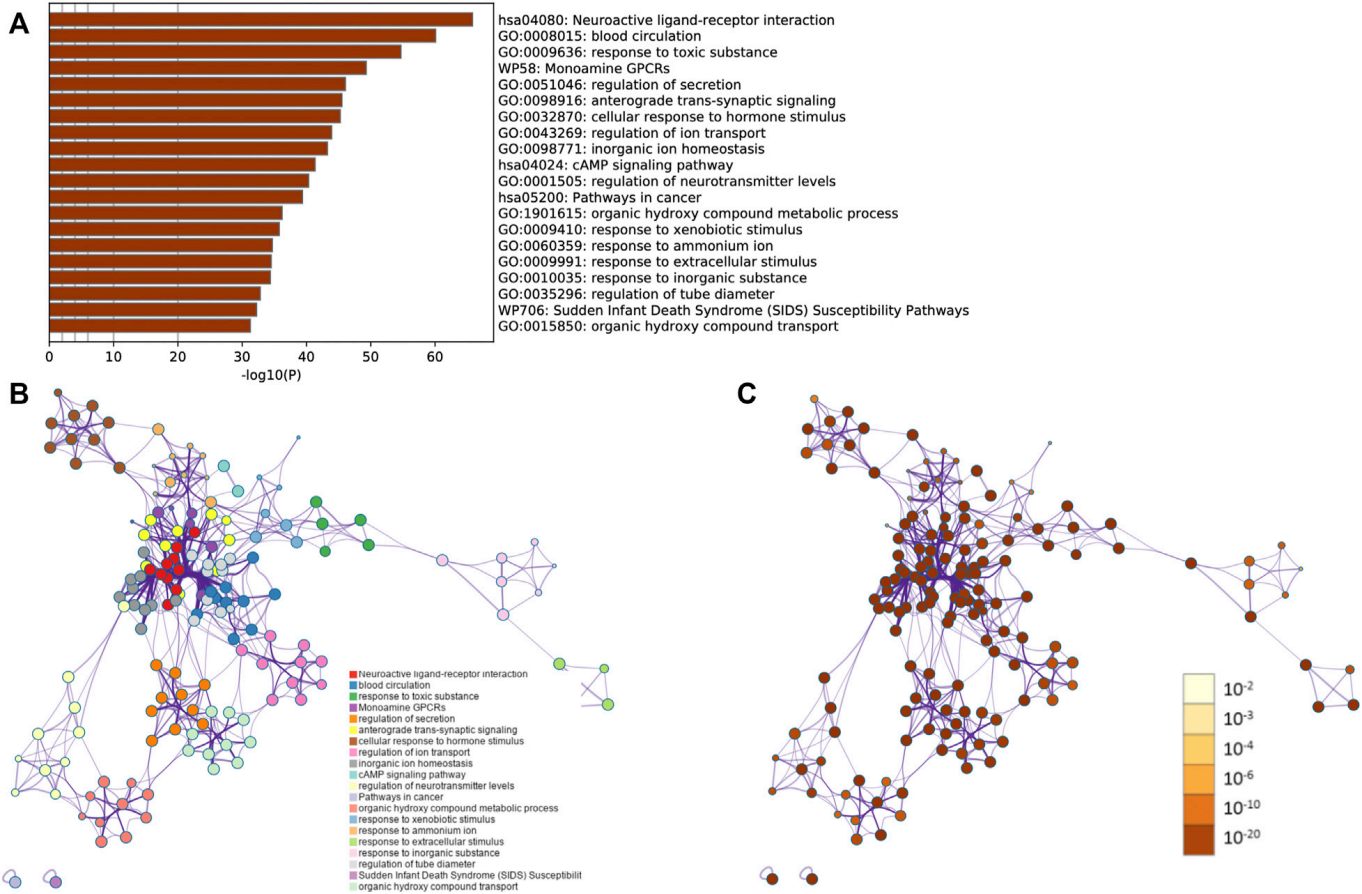
Gene annotation and the enrichment analysis.

To illuminate potential functions of these potential targets (328 target genes after intersection), gene annotation and a function enrichment analysis has been performed. The top twenty representative terms were displayed (Figure 3A). Furthermore, a network based on subsets was constructed, with a condition of similarity >0.3 (Figures 3B,C). Specifically, the genes enriched in SJHY formula for the treatment of diabetic wound healing were associated with the neuroactive ligand–receptor interaction and the cAMP signaling pathway, along with the pathway in cancer.

### 3.4 PPI and Kernel Cluster Network Construction

**FIGURE 4:**
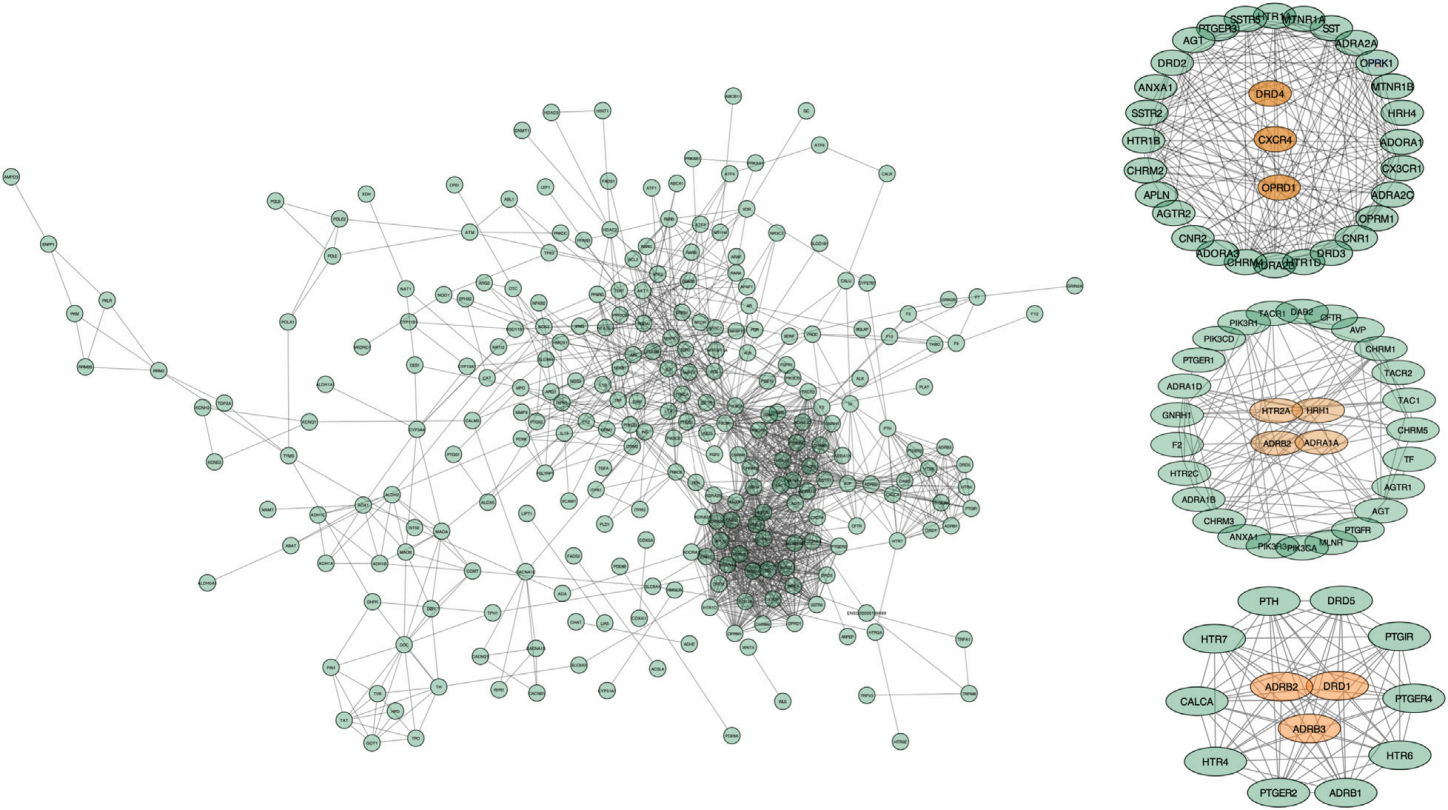
Modular pharmacology and the kernel cluster analysis based on the ClusterONE algorithm. The points represent genes, and the edges represent protein–protein interaction. The points in orange color represent the kernel genes, and the green points represent the genes that interacted with kernel genes.

In order to further investigate the hub genes, a new modular network was established according to the ClusterONE algorithm for determining the kernel therapeutic targets (Figures 4A,B). Based on the conditions, a total of three clusters were selected for a subsequent analysis (Table 2). Following the filter condition of each cluster, CXCR4, DRD4, and OPRD1could be considered as kernel targets. ADRB2 reached the highest degree of freedom in Cluster 2, followed by HRH1, HTR2A, and ADRA1A. Except for ADRB2 and DRD1, Cluster 3 contained ADRB3. Taken together, these results suggested that those kernel targets could be considered as the kernel targets of SJHY formula–treated diabetic wound healing (Table 3).

**TABLE 2:** Modular analysis using the ClusterONE algorithm.

Cluster	Size	Density	Internal weight	External weight	Quality	*p*-value	Members
*1	31	1	465	102	0.82	0	SST CHRM2 AGT AGTR2 CXCR4 OPRM1 APLN ANXA1 SSTR2 SSTR5 OPRK1 OPRD1 DRD3 HTR1D HTR1B DRD2 PTGER3 HTR1A MTNR1A MTNR1B HRH4 DRD4 ADRA2C CHRM4 CNR2 CNR1 CX3CR1 ADRA2B ADORA3 ADRA2A ADORA1
*2	29	0.7512	305	193	0.612	2.20E-06	PIK3CA PIK3R1 F2 PIK3R3 TAC1 TF PIK3CD DAB2 TACR1 CFTR AVP ADRB2 AGT AGTR1 CHRM1 PTGER1 HTR2C HTR2A HRH1 CHRM5 ANXA1 TACR2 PTGFR GNRH1 CHRM3 MLNR ADRA1D ADRA1B ADRA1A
*3	13	1	78	33	0.703	3.36E-05	HTR7 PTH DRD1 HTR4 DRD5 PTGER4 PTGIR HTR6 PTGER2 ADRB3 CALCA ADRB1 ADRB2
*4	10	0.5556	25	8	0.758	0.00023215	HPD TAT TH DHFR PAH TPH1 TYR GOT1 TPO DDC
*5	11	0.5091	28	17	0.622	0.0006195	DDC MAOB MAOA DBH COMT ADH1B ADH1C AOX1 ALDH2 ADH1A ABAT

PPI, protein–protein interaction.

**TABLE 3:** Hub genes based on the modular analysis.

Symbol	Uniprot ID	Description	Relevant compound
CXCR4	P61073	C-X-C chemokine receptor type 4	Astragaloside
			Astramembrannin
			Sucrose
OPRD1	P41143	Delta-type opioid receptor	Lobelanidine
			Oxyphyllacinol
DRD4	P21917	D (4) dopamine receptor	Danshensu
			Dauricine
			P-Cymen-2-Ol
			Serotonine
HTR2A	P28223	5-hydroxytryptamine receptor 2A	Dauricine
			O9-Angeloylretronecine
			Serotonine
HRH1	P35367	Histamine H1 receptor	Lobelanidine
			Serotonine
ADRB2	P07550	Beta-2 adrenergic receptor	Danshensu
			N-Methylephedrine
			Serotonine
ADRA1A	P35348	Alpha-1A adrenergic receptor	Silicon
			Serotonine
			N-Methylephedrine
			Danshensu
			2-Methyl Cardol
ADRB3	P13945	Beta-3 adrenergic receptor	Serotonine
DRD1	P21728	D (1A) dopamine receptor	Dauricine
			N-Methylephedrine
			P-Cymen-2-Ol
			Serotonine

**FIGURE 5:**
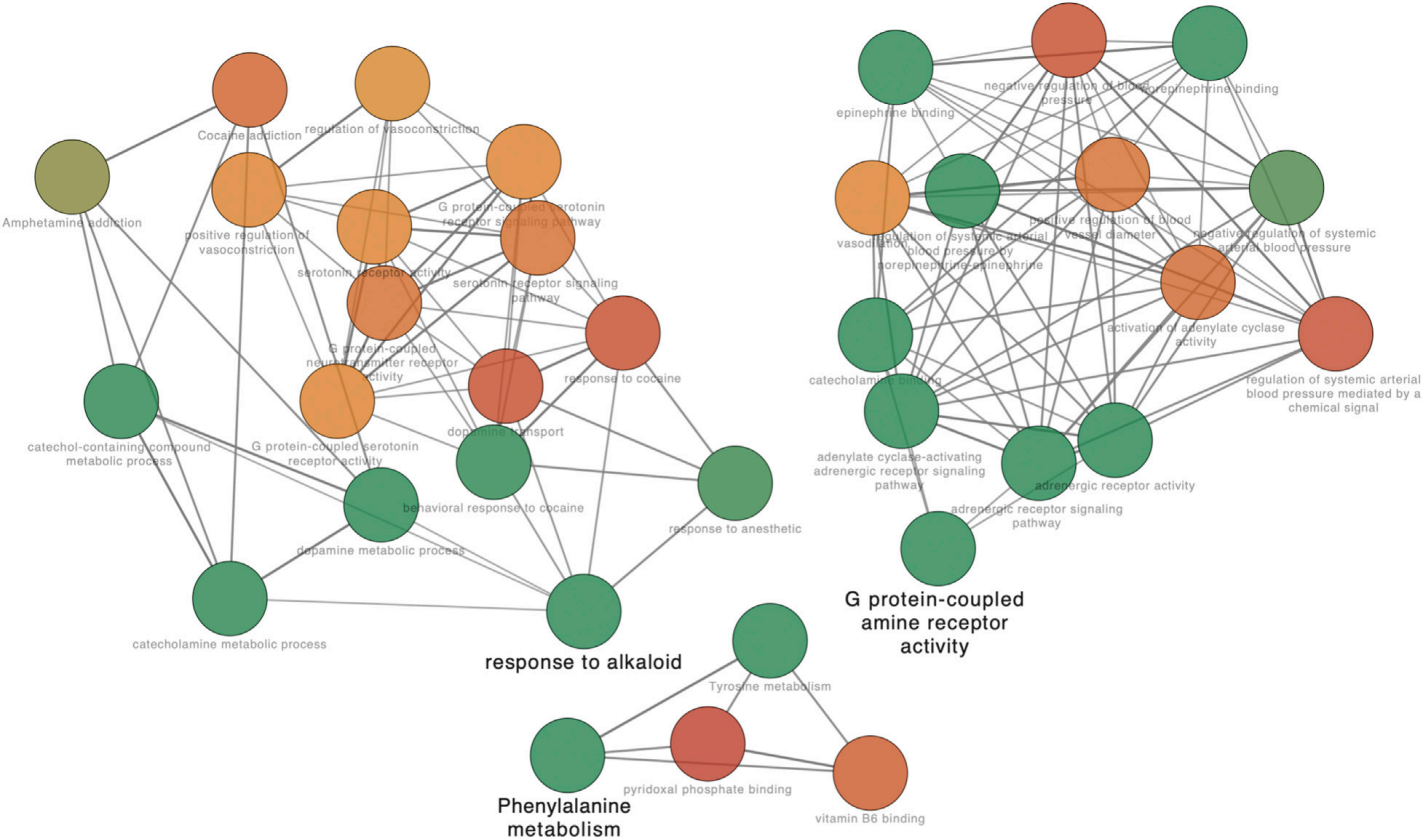
GO network analysis based on kernel targets. The nodes in different colors represent different enrichment degree. Red color means high enriched GO terms, and green color means low enrichment degree.

**FIGURE 6:**
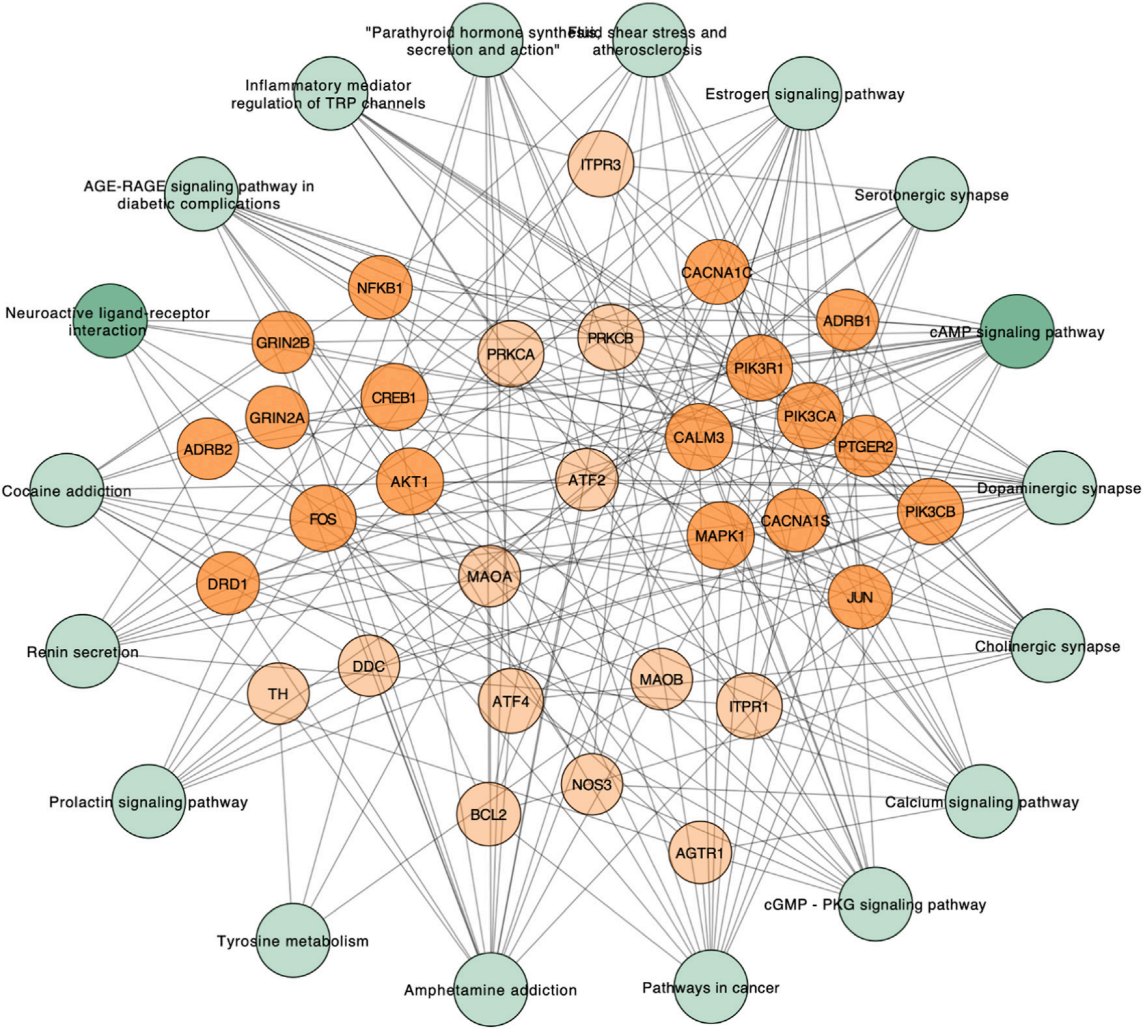
KEGG–target network analysis based on kernel pathways.

Further analysis of the GO network was conducted on Cytoscape software, and the data revealed the main outcomes of GO characterization: vitamin B6 binding, response to alkaloid, and G protein–coupled amine receptor activity (Figure 5). To further investigate the regulatory relationship between the targets, we have drawn a pathway–target network analysis using screening conditions: BC > Avg (BC), CC > Avg (CC), and De > Avg (De). As Figure 6, it manifested that the neuroactive ligand–receptor interaction (Supplementary Figure S2) and the cAMP signaling pathway (Supplementary Figure S3) could be considered as the kernel pathways of the SJHY formula–treated diabetic wound healing.

### 3.5 Molecular Docking

**FIGURE 7:**
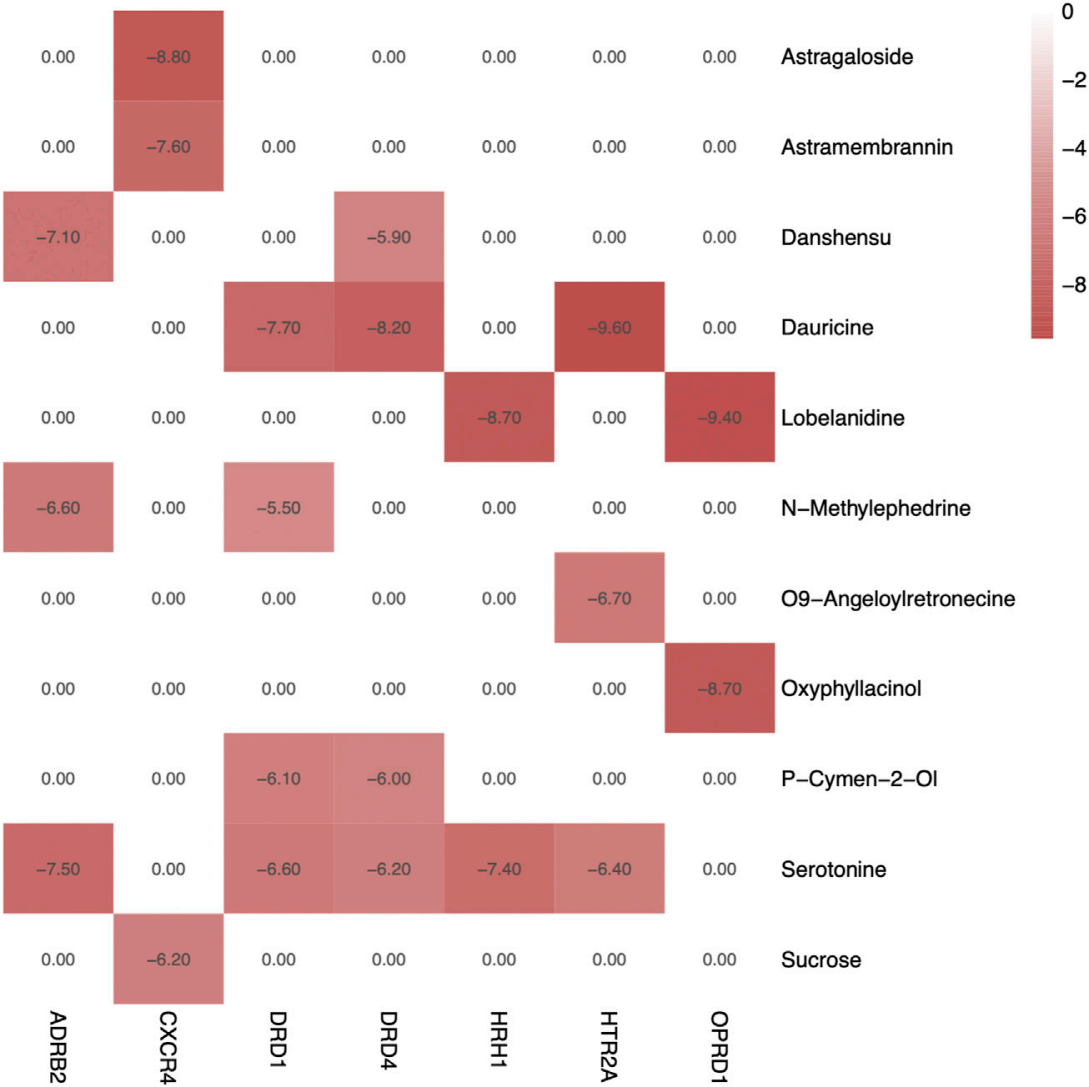
Heatmap of binding energy of compounds and targets. Color of red represents high BE values, and white and pink colors mean a low value of binding affinity.

**FIGURE 8:**
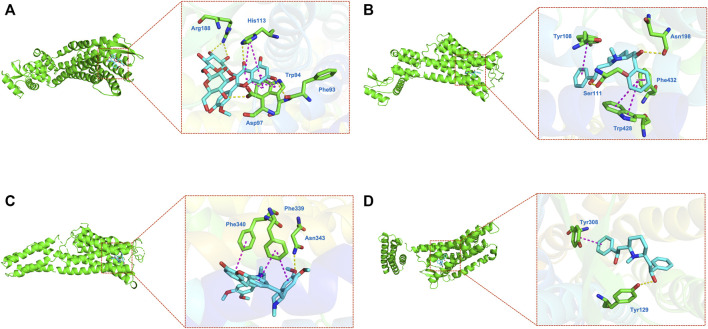
Molecular docking on hub genes. **(A)** CXCR4–astragaloside; **(B**, HRH1–lobelanidine; **(C)** HTR2A–dauricine; **(D)** OPRD1–lobelanidine.

Molecular docking was adopted for predicting bond conformations and free binding energies in the current study. The relevant results in binding energy (BE) of the small molecule and macromolecule were considered as an evaluation index. BE values less than −7 kcal/mol were considered to represent significant binding affinity, while BE < −8.5 kcal/mol were chosen for molecular docking. The results of the binding energy computations can be obtained from Table 4, while the correlations analysis with bivariate correlations can be obtained from Figure 7. Based on the evidences from molecular docking, we considered that the structure of CXCR4–Astragaloside (BE = −8.8 kcal/mol; Figure 8A), HRH1–Lobelanidine (BE = −8.7 kcal/mol; Figure 8B), HTR2A–Dauricine (BE = −9.8 kcal/mol; Figure 8C), and OPRD1–Lobelanidine (BE = −9.4 kcal/mol; Figure 8D) were most stable, while DRD1, DRD4, and ADRB2 were also proposed as kernel targets of SJHY formula–treated diabetic wound healing.

**TABLE 4:** Molecular docking.

Ligand	Genes	PDB ID	Affinity kcal/mol
Danshensu	ADRB2	2RH1	−7.1
N-Methylephedrine	ADRB2		−6.6
Serotonin	ADRB2		−7.5
Astragaloside	CXCR4	3OE8	−8.8
Astramembrannin	CXCR4		−7.6
Sucrose	CXCR4		−6.2
Dauricine	DRD1	7CRH	−7.7
N-Methylephedrine	DRD1		−5.5
P-Cymen-2-Ol	DRD1		−6.1
Serotonin	DRD1		−6.6
Danshensu	DRD4	6IQL	−5.9
Dauricine	DRD4		−8.2
P-Cymen-2-Ol	DRD4		−6
Serotonin	DRD4		−6.2
Lobelanidine	HRH1	3RZE	−8.7
Serotonin	HRH1		−7.4
Dauricine	HTR2A	6W4H	−9.8
O9-Angeloylretronecine	HTR2A		−6.7
Serotonin	HTR2A		−6.4
Lobelanidine	OPRD1	4N6H	−9.4
Oxyphyllacinol	OPRD1		−8.7

### 3.6 Experimental Verification

#### 3.6.1 Quantitative Analysis of SJHY Formula by LC-MS/MS

**FIGURE 9:**
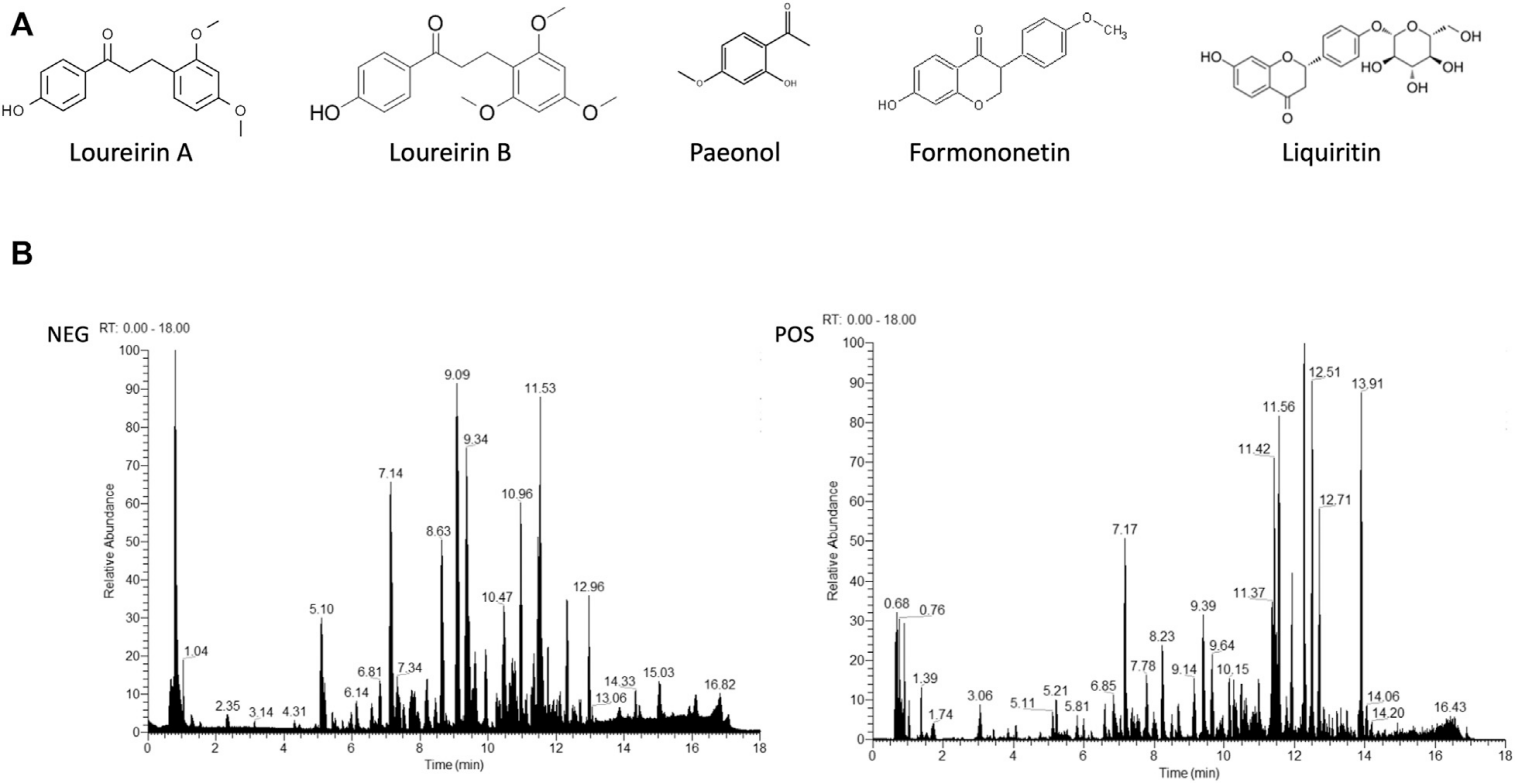
Analysis of components of SJHY formula. **(A)** Chemical structure of loureirin A, loureirin B, paeonol, formononetin, and liquiritin, respectively; **(B)** Total ion chromatogram of SJHY formula separated by a positive and negative ion mode.

LC-MS/MS analysis was performed to establish the quality control of SJHY formula by identifying and quantifying ingredients involving loureirin A, loureirin B, paeonol, formononetin, and liquiritin. The structure of the aforementioned compounds is displayed in Figure 9A, and the general ion flow chromatographic of SJHY formula is shown in Figure 9B. The content of loureirin A, loureirin B, paeonol, formononetin, and liquiritin in the SJHY formula was 113.41 ug/ml, 129.39 ug/ml, 21.82 ug/ml, 13.99 ug/ml, and 5.00 ug/ml, respectively (Table 5).

**TABLE 5:** Quantification of components from SJHY formula.

Component name	Loureirin A	Loureirin B	Paeonol	Formononetin	Liquiritin
Content in SJHY formula (ug/ml)	113.41	129.39	21.82	13.99	5.00

#### 3.6.2 SJHY Formula Regulated mRNA Expression of Kernel Genes

**FIGURE 10:**
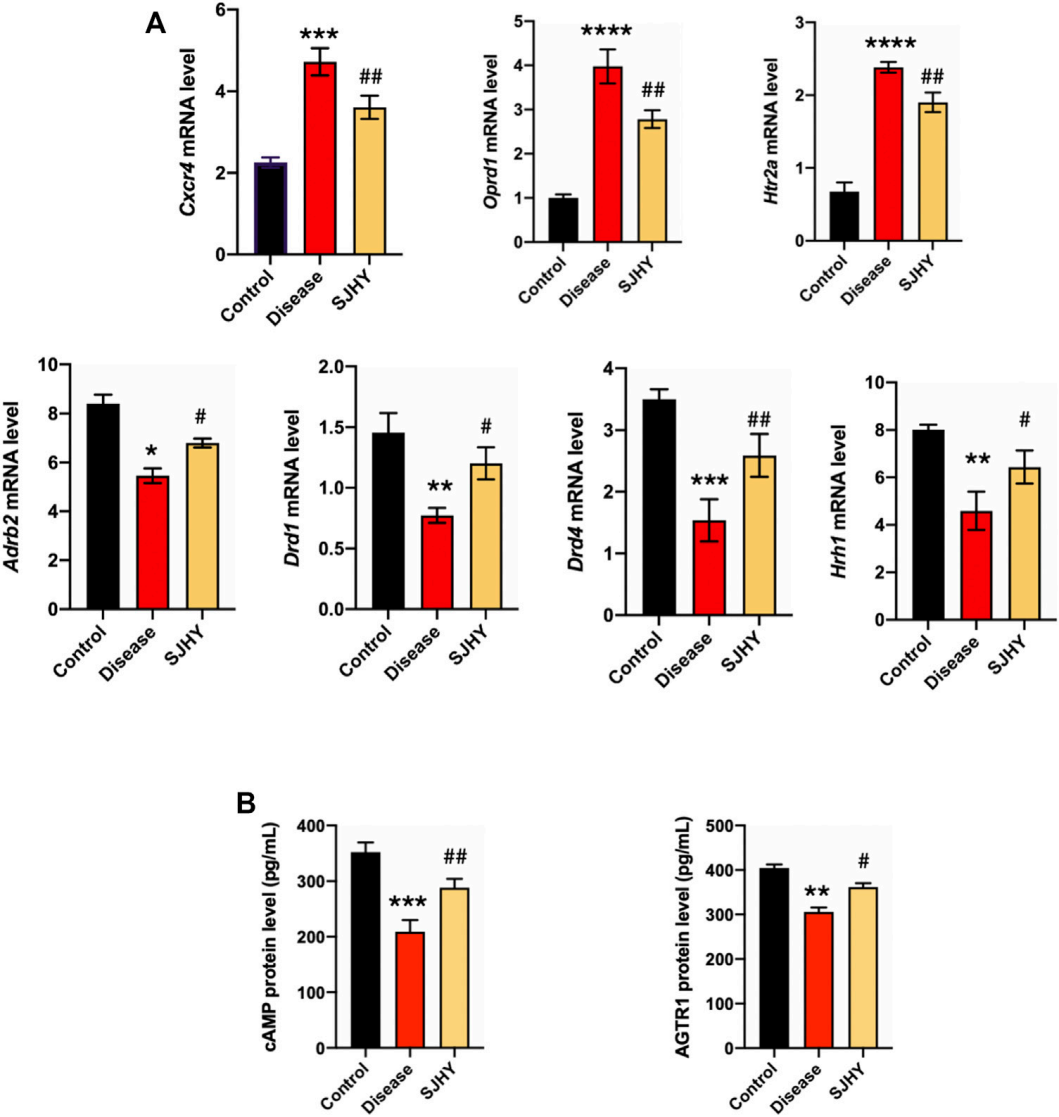
Experiment validation of hub genes and kernel pathways on day 9 after punching. **(A)** Detection of expression of hub genes using qPCR; **(B)** Validation of kernel pathways using ELISA. ^#^ *p* < 0.05, ^##^ *p* < 0.01, compared with the control group. *p < 0.05, **p < 0.01, ***p < 0.001, **** *p* < 0.0001 compared with the disease group.

To verify the kernel network, the mRNA levels of the target genes was quantified by real-time PCR, and all seven genes reached statistical significance. The results of qPCR showed that the SJHY formula downregulated *Cxcr4*, *Oprd1*, and *Htr2a* with statistical significance, on comparison with the disease group. At the same time, the mRNA expression levels of *Adrb2*, *Drd1*, *Drd4*, and *Hrh1* were significantly increased. Together these results provided important insights that the mRNA expression level of hub genes might help evaluate the efficacy of SJHY formula for the treatment of diabetic wound healing (Figure 10A).

#### 3.6.3 SJHY Formula Increased Protein Expressed Levels of Kernel Pathway–Related Genes in Mice

The ELISA was adopted to evaluate the expression of cAMP and AGTR1, neuroactive ligand–receptor interaction, and cAMP signaling pathway–related genes in the tissue homogenate sample after SJHY formula treatment, and these proteins showed that the expression of cAMP and AGTR1 were increased with statistically significance (Figure 10B).

## 4 Discussion

Wound healing is a major complication of diabetes which usually leads to limb loss or disability (den Dekker et al., 2019). Currently, the Food and Drug Administration (FDA) declared for the clinical use of a platelet-derived growth factor, which was the only drug for the treatment of diabetic wound. Additionally, alternative therapies such as SJHY formula, could improve clinical response rate and shorten the clinical course by 2–3 days compared with Western medicine treatment, which are increasingly getting clinical applications worldwide. The therapeutic effect of TCM on diabetic wound healing is mainly through the interaction between multiple molecules.

We have previously demonstrated the therapeutic effects of SJHY formula on diabetic wound healing (Li et al., 2011; Kuai et al., 2018). Notably, the LC-MS/MS analysis was utilized to identify and quantify the compounds of SJHY formula, involving loureirin A, loureirin B, paeonol, formononetin, and liquiritin (Figure 9; Table 5). Loureirin A play an active role in wound healing by promoting hair follicle stem cells to repair skin epidermis (Li et al., 2018). Interestingly, loureirin B may downregulate the mRNA and protein levels of several fibrosis-related molecules and suppress the proliferation of hypertrophic scar (HS) fibroblasts, thus reducing scar formation (Bai et al., 2015). Paeonol, a compound that possesses anti-inflammatory, analgesic, antioxidant, and anti-allergic properties, has been proved to treat inflammation-related diseases, allergies, and cancer (Liu et al., 2014). Furthermore, formononetin inhibits NO production, inflammatory infiltration, and improve blood microcirculation, resulting in accelerated wound healing (Huh et al., 2011; Lai et al., 2013). As for liquiritin, it has been applied to treat against skin inflammation, various cancers, and digestive disorders by reducing inflammation, oxidative stress, apoptosis, and metabolic alterations (Xiong et al., 2021; Han et al., 2019). Taken together, these evidences may explain the clinical phenomenon that SJHY formula ameliorates retarded wound healing of diabetes and simultaneously reduces scar formation.

In the present study, the ClusterONE algorithm and molecular docking were applied to the compound prescription of TCM-treated diabetic wound healing. According to the networks, 529 components of SJHY formula were selected by the TCMSP database. Furthermore, we obtained 659 corresponding targets. Moreover, disease-related targets were determined using the five databases. After the intersection of drug targets and disease targets, 328 targets were obtained. As the modular network reveals and molecular docking experiment validates, we inferred that the following proteins could be considered as hub genes of SJHY formula: OPRD1, CXCR4, HTR2A, HRH1, DRD1, DRD4, and ADRB2.

The diabetic wounds have multiple factors, broadly divided into three categories: neuropathy, chronic inflammation, and angiopathy (Moura et al., 2019). Peripheral neuropathy is routinely used in risk assessment, presently in roughly half of non-healing wound cases and neurological evaluation (Moura et al., 2019). The present study indicated that SJHY formula might regulate multiple neurotransmitters levels to treat diabetic wound healing. OPRD1, a target of many regulators to modulate its neurotransmission for the role in neurosecretion, was significantly upregulated following SJHY formula. These results were in keeping with previous animal experiment studies, which indicated that OPRD1 was significantly regulated by nerve damage in the ganglion, and upregulated on the 4th day after the injury (Korczeniewska et al., 2020). Besides the decreased pain sensation, neuropathy also decreases neuropeptide expression (da Silva et al., 2010), which could regulating inflammation in the process of wound healing (Curtis and Radek, 2012).

Chronic inflammation is considered as a center link in the progression of diabetic wound healing disease (Moura et al., 2019). C-X-C motif chemokine receptor 4 (CXCR4), thought to serve a critical role in the regulation of inflammation and cancer biology, has gained a lot of attention in recent years (Kircher et al., 2018). The expression of CXCR4 was upregulated in the blood vessels, macrophages, and skin tissue of K14-VEGF-A induced mice (Zgraggen et al., 2014), and indicated the pivotal role in early inflammation (Konrad et al., 2017). CXCR4 was identified as a hub gene of SJHY formula with one of the lowest binding energy values in molecular docking, while qPCR verified that *Cxcr4* was decreased after treatments. These results were consistent with those in previous studies.

The results on mRNA expression of *Htr2a* reflected those in the previous study of Yang et al., who had also found that accumulation of macrophages and neutrophils along with increased levels of inflammatory cytokines, were associated with inflammation (Yang et al., 2020). Meanwhile, the absence of 5-HT suppressed hepatic lipid load and the expression of inflammatory factors, including TNF-α, IL-6, and MCP1 (Wang L. et al., 2020). Another animal experiment also validated that HTR2A was upregulated significantly after inflammation/pain in the dorsal root ganglion following disc puncture in rats (Fujioka et al., 2016).

Histamine and histamine receptors (HRHs) have been confirmed as a kernel molecule in inflammation (Tanaka et al., 2016). Histamine promotes function of keratinocytes in inflammation, and keratinocytes receptors get involved in histamine-induced inflammation, predominantly mediated by HRH1 (Gutowska-Owsiak et al., 2014). Besides, adrenergic receptor (ADR)-agonists have indicated anti-inflammatory characteristics in immune or non-immune cell among various tissues. The present study confirmed several previous findings. Their anti-inflammatory properties are primarily mediated by overexpression of ADRB2 (Dorotea and Ha, 2021). The previous reports are in accordance with our experiment results.

Dopamine receptors are expressed in many immune cells and suppress inflammations (Liu et al., 2021). Our results of DRD1 and DRD4 corroborate the findings of a great deal of the previous study. One previous study reported that it inhibited NLRP3 inflammasome activation *via* DRD1, which negatively regulated NLRP3 inflammasome by cAMP, promoting ubiquitination of NLRP3 together with its degradation. Furthermore, it has been observed that DRD1 prevented NLRP3 inflammasome–dependent inflammation *in vivo* (Diaz-Espinosa et al., 2020). Further studies found that DRD4 downregulated the expression of cAMP, which further inhibited the PKA/p38 signaling pathway and downregulated the inflammation factors in a mouse model (Liu et al., 2021). The evidences on qPCR attributed that SJHY formula upregulated the expression of DRD1 and DRD4.

The neuroactive ligand–receptor interaction contained a few pairs, including HTR, DRD, and ADR, which had been considered as hub genes of SJHY formula treated diabetic wound healing as well as played a regulatory role in peripheral nerve disease and chronic inflammation. Besides, the current study detected protein expression of AGTR1, and the result validated that SJHY formula could significantly regulate a relevant neuroactive ligand–receptor interaction.

Another kernel pathway, the cAMP signaling pathway, has been reported in previous studies that it has a role in proliferation, inflammation, and migration, which are present on the progression through distinct yet overlapping phases of wound healing (Boniakowski et al., 2017). cAMP has a dual effect on cell proliferation in the early G0 or G1 phase. Second, cAMP binds to NLRP3 and promotes its ubiquitination together with degradation through the E3 ubiquitin ligase MARCH7. Besides, an increase of cyclic adenosine monophosphate in adult neurons will cause them to grow like newborn neurons in the presence of protein myelin-associated glycoprotein. Consistent with the previous validation, the protein expression level of cAMP was significantly reduced following SJHY formula in the present study, which suggested that the cAMP signaling pathway could be the kernel pathways of SJHY formula treated diabetic wound healing.

Nevertheless, the current study also has some limitations. First, the evidences of molecular docking provided several pairs of TCM ingredients and target, which required further inquiry. Second, genetic function and rescue experiments on hub genes and kernel pathways were required in future research. Third, clinical sample validation should be further conducted.

## 5 Conclusion

The current study used modular pharmacology analysis to identify the hub genes and pathways of SJHY formula–treated diabetic wound healing. The present study proved that SJHY formula downregulated the mRNA expression of *Cxcr4*, *Oprd1*, and *Htr2a*, while upregulated *Adrb2*, *Drd*, *Drd4*, and *Hrh1*. Furthermore, kernel cluster enrichment analysis indicated that the SJHY formula could regulate the neuroactive ligand–receptor interaction and the cAMP signaling pathway to ameliorate skin lesions of diabetic wound healing–like mice model.

## Data Availability

The raw data supporting the conclusions of this article will be made available by the authors, without undue reservation, to any qualified researcher.

## Supplementary Material

Supplementary Figure 1 Approval of animal experiment ethic application.

Supplementary Figure 2 Map of the neuroactive ligand–receptor interaction.

Supplementary Figure 3 Map of the cAMP signaling pathway.

## Abbreviations

GO, gene ontology; KEGG, Kyoto Encyclopedia of Genes and Genomes; LC-MS/MS, Liquid chromatography tandem–mass spectrometer; OMIM, Online Mendelian Inheritance in Man; PPI, protein–protein interaction; SJHY, Sheng-JiHua-Yu; SPF, specific pathogen-free; TCM, traditional Chinese medicine; TCMSP, traditional Chinese medicine systems pharmacology database and analysis platform; TTD, Therapeutic Target Database.
