# Supplementary material for: The Identification of the Biomarkers of Sheng-Ji Hua-Yu Formula Treated Diabetic Wound Healing Using Modular Pharmacology
Source: Front Pharmacol. 2021 Nov 16;12:726158. doi: 10.3389/fphar.2021.726158 (PMC8636748; doi:10.3389/fphar.2021.726158)
Supplement: Supplementary file 1 [file Table1.DOCX]

Table S1. Ingredients of SJHY formula

| Main composition | English translation | Plant part | Amount (g) |
| --- | --- | --- | --- |
| Radix Astragali | Astragalus membranceus (Fisch.) Bunge., Radix | Radix | 60.05 |
| Salviae Miltiorrhizae Radix et Rhizoma | Salvia miltiorrhiza Bge., Rhizoma | Rhizoma | 15.03 |
| Rhei Radix et Rhizoma | Rheum palmatum L., Rhizoma | Rhizoma | 15.12 |
| Draconis Sanguis | Daemonorops draco Bl., Resin | Resin | 10.06 |
| Arnebiae Radix | Arnebia euchroma (Royle.) Johnst., Radix | Radix | 30.16 |
| Angelica dahurica | Angelica dahurica (Hoffm.) Benth. & Hook.f. ex Radix Franch. & Sav., Radix | Radix | 30.35 |
| Margaritifera Concha | Hyriopsis cumingii (Lea.) Draconis Sanguis | - | 30.10 |
| Calamina | Calamina | - | 30.05 |
