# Supplementary figures and images for: The Identification of the Biomarkers of Sheng-Ji Hua-Yu Formula Treated Diabetic Wound Healing Using Modular Pharmacology
Source: Front Pharmacol. 2021 Nov 16;12:726158. doi: 10.3389/fphar.2021.726158 (PMC8636748; doi:10.3389/fphar.2021.726158)

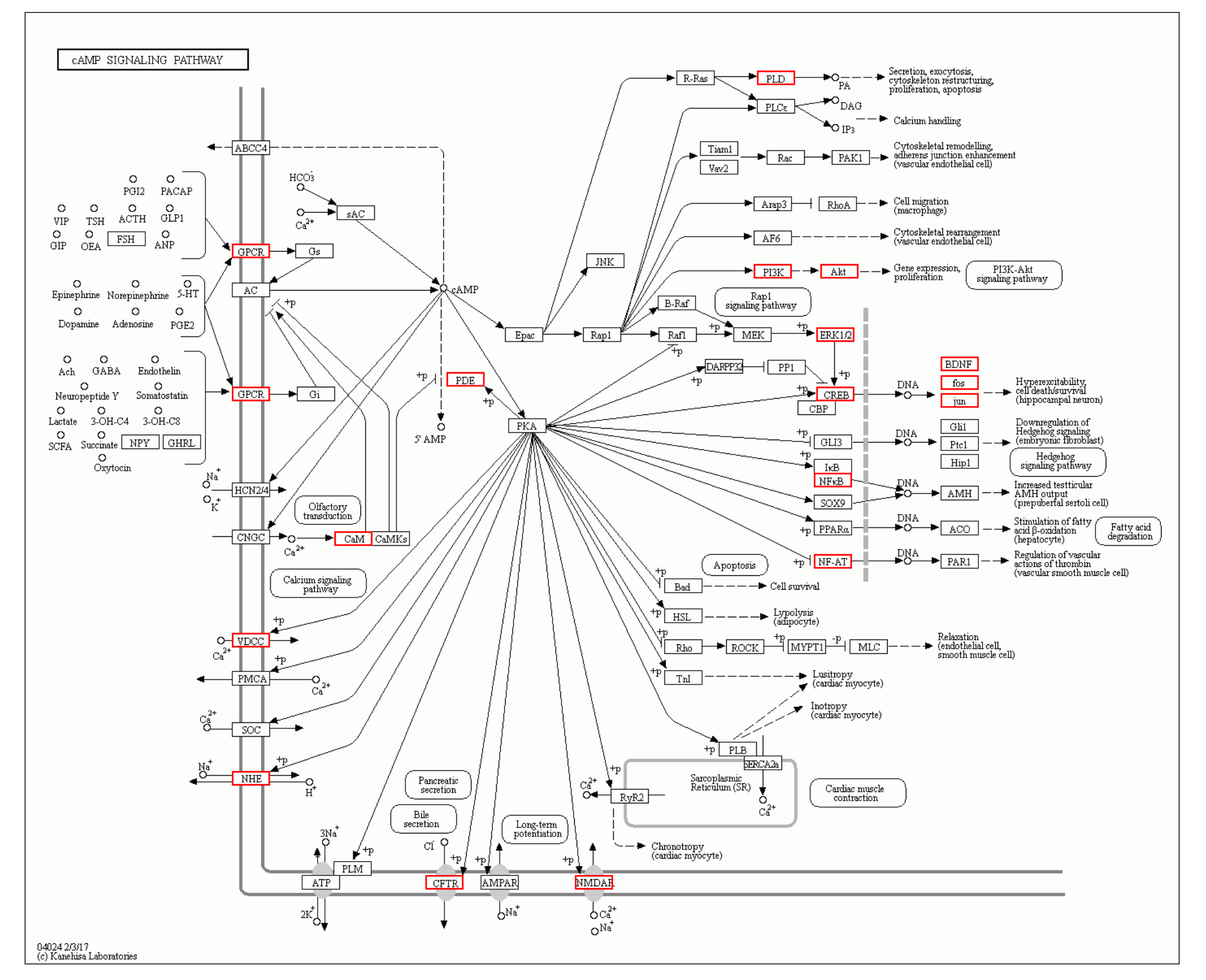

Supplement: Supplementary file 2 [file Image3.TIF]

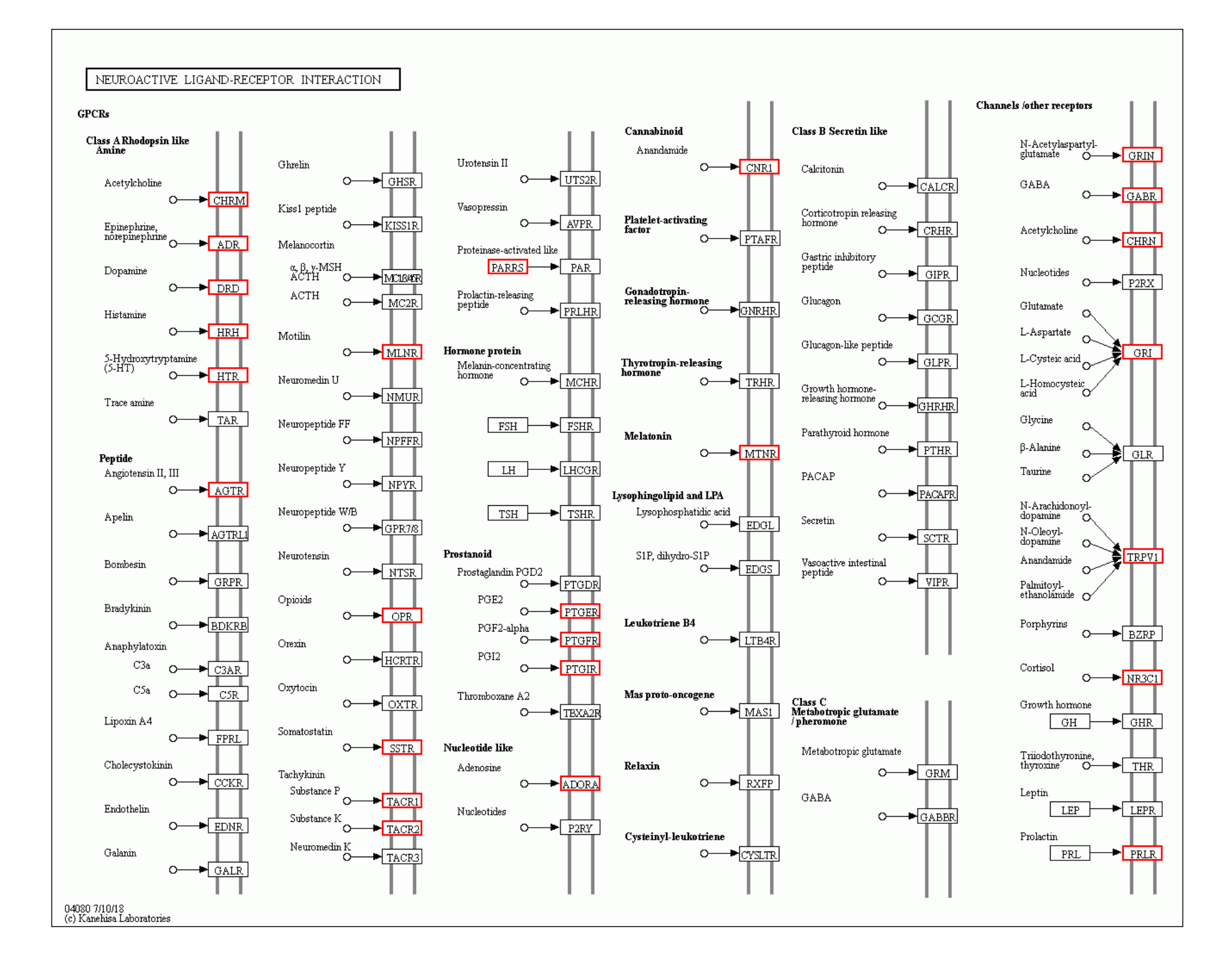

Supplement: Supplementary file 3 [file Image2.TIF]

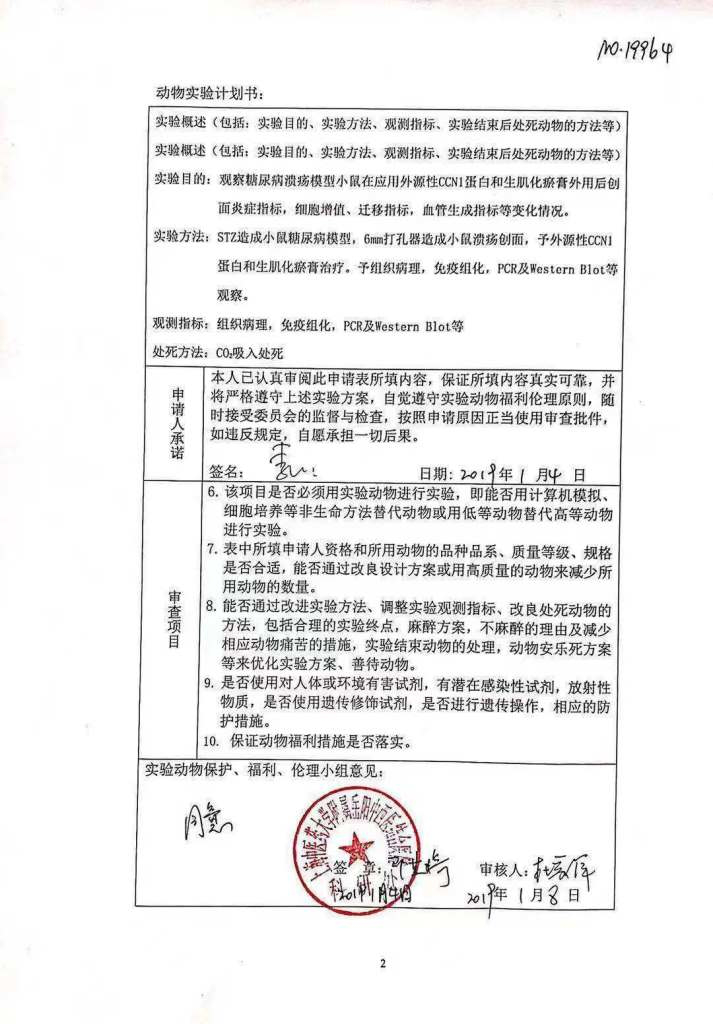

Supplement: Supplementary file 4 [file Image1.TIF]
