# Supplementary material for: The Identification of the Biomarkers of Sheng-Ji Hua-Yu Formula Treated Diabetic Wound Healing Using Modular Pharmacology
Source: Front Pharmacol. 2021 Nov 16;12:726158. doi: 10.3389/fphar.2021.726158 (PMC8636748; doi:10.3389/fphar.2021.726158)
Supplement: Supplementary file 6 [file Table2.DOCX]

Table S2. Primers used for qPCR

| Name | Forward | Reverse | Amplicon (bp) |
| --- | --- | --- | --- |
| *mCxcr4* | ACTACACCGAGGAAATGGGC | AGGTGCAGCCTGTACTTGTC | 208 |
| *mDrd1* | ATCCTTTCCCCAAACGCACC | GCAGCTCTCCAAACGCCTTG | 186 |
| *mDrd4* | TGGTGGTCGGGGCCTT | GTAGATGACGGGGTTGAGGG | 152 |
| *mOprd1* | CGCACGGTGGAGAGGGAC | CTTCATCTTAGTGTACCGGACGA | 283 |
| *mHtr2a* | TCTCACCGTCGTGTCTCTCC | GGCCACCGGTACCCATACA | 244 |
| *mHrh1* | AAGCCCATCATGGAGAAGACC | CAGTTATGGCTCACTCCCTTT | 135 |
| *mAdrb2* | TGAAGGCCTATGGGAATGGC | AGGCACAGTACCTTGATGGC | 146 |
| *mAdrb3* | TCAACCCGCTCATCTACTGC | CCCAAGAAGCCCCGTCG | 185 |
| *mAdra1a* | GACAGCGGTTCAGGAGCTTA | TTTGACCGCGTCCACCTGAA | 208 |
